# Commentary: Sure I'm Sure: Prefrontal Oscillations Support Metacognitive Monitoring of Decision Making

**DOI:** 10.3389/fpsyg.2017.02331

**Published:** 2018-01-09

**Authors:** Hamid Ostad Rahimi, Farzaneh Rahmani

**Affiliations:** ^1^Neuroimaging Network (NIN), Universal Scientific Education and Research Network (USERN), Tehran, Iran; ^2^Students Scientific Research Center, Tehran University of Medical Sciences, Tehran, Iran

**Keywords:** prefrontal cortex, metacognition, oscillations, electroencephalography, decision making

Metacognition, as defined as monitoring and controlling of the decision-making process in the brain (Fleming and Dolan, 2012), plays a major role in adjustment of the ongoing behavior of a high order organism, most importantly in the mammalian brain. Metacognition helps to determine in a roadmap, next, and best moves in reaction to external stimuli when external feedback is not immediately available. Little is known about the underlying neural mechanisms of metacognition, and it remains controversial whether different neural circuits are involved in processing information used in first-order decisions vs. those in metacognitive ones. Some propose that similar brain process is engaged in both kinds of processes (Kiani and Shadlen, 2009). This hypothesis suggests that same information, regarding quality and quantity, contributes to form either a first order or a second order (metacognitive) decision, while distinct behaviors are suggestive of different underlying information sources (Cleeremans et al., 2007). En route to give a proper explanation for this theory, it has been proposed that various levels of available information lead to different decisions in first and second order decision making, by emphasizing the role of noise accrual and signal decay that occur within metacognitive system networks (Pleskac and Busemeyer, 2010). Trial-by-trial choosing tasks are widely used to mimic first-order decision making experiments and permit similar electrophysiological cortical oscillatory dynamics to be captured. By all above, it remains unclear how oscillations relate to second-order decision making. Recently, Wokke et al. (2016) suggested that a significant electrophysiological oscillatory change-in-pattern happens with metacognitive decision making. They demonstrated the idea that certain oscillatory pathways in the brain can reflect differences between first-order and metacognitive task performance.

In the study by Wokke et al. (2016), participants had to make a diagnosis after being presented with fictitious patient data in the form of an intricate pattern of colored moving dots in different sizes. Participants were entered in a study, in which no background information was useful to identify the patient's diagnosis. During each trial, one color, one size, and one motion direction were indicative of the correct illness situation i.e., observing more than some dots with a particular color moving in a previously convened direction indicates “patient” diagnosis. The experiments were designed in a way that allowed researchers to record EEG fluctuation in each single trial in the case of accuracy, metacognitive adequacy and reasoning strategy. After each trial, participants were asked to choose the reasoning strategy they used for their diagnosis that could be “chancy choosing,” “uncertain,” or “rational” diagnosis. Next, they rated their certainty about each single trial, with all the process going on while recording EEG signals with the purpose of revealing the relationship between accuracy in diagnosis, self-selected strategy of choice and metacognitive adequacy, extracted from answers with fluctuations in EEG bands. Multiple regression analysis allowed assessment of components of both first-order and metacognitive decision separately.

Here, Wokke et al. (2016) reported three possible models of the differences between first-order and metacognitive decision making based on brain activity indicators. They found a positive correlation between prefrontal theta band activity in EEG signals and metacognitive performance that was not explicable by first-order performance or the variety stimulus parameters. We know that theta band activities are related to accumulation and integration of pieces of evidence in the brain (van Vugt et al., 2012) and that increased task accuracy is related to decreased beta band activity in motor areas of the brain. Thus, the beta band activity reflects motor choices, and lower uncertainty in motor action (Donner et al., 2007). These findings are consistent with the presence of a hierarchical model and second order network that learns to interpret contingencies in first-order networks to evaluate which activity patterns result in successful decision making.

Interestingly, metacognitive adequacy could not be predicted by participant-reported reasoning strategy, while the final first-order task performance, was significantly associated with the reasoning strategy. Similarly, there was no significant difference in EEG bands in different strategies that participants used, an early clue showing the different participation of information certainty, in first-order decision making and the metacognitive one. Various components that contribute to first-order decision making (size, color, and motion) have a rather weak contribution in the metacognitive task performance as claimed by Pleskac and his colleagues (Pleskac and Busemeyer, 2010). It appeared that the size of dots, but not the other two characteristics, might affect subjective (metacognitive) task performance. They revealed that information used during this processes differ by their ultimate quality and that a second order processing method, emphasizing on limited characteristics, is crucial for a successful decision making.

Theta band activities are thought to facilitate the connection between prefrontal cortex and task-related networks. Interestingly, as previous studies showed lesions in prefrontal cortex lead to metacognitive deficits, Wokke et al. (2016) research also indicate that adaptive decision making and theta oscillations are interrelated. The authors' data sharing is valuable, and the data they provided could be an inspiration for other researchers and directs the related fields forward.

## Author contributions

HO: Conceptualized and drafted the manuscript; FR: helped in drafting and edition.

### Conflict of interest statement

The authors declare that the research was conducted in the absence of any commercial or financial relationships that could be construed as a potential conflict of interest.
